# Microsurgical treatment of deep maxillary Class II trifurcation defects combining root resection and guided tissue regeneration: a case series

**DOI:** 10.1038/s41415-026-9527-8

**Published:** 2026-05-08

**Authors:** Pedro Franco Ferreira, Milenna Silva Fuly, Márcio Fernando de Moraes Grisi, Ana Carolina Fragoso Motta

**Affiliations:** 373847710716281431521 Department of Oral & Maxillofacial Surgery, and Periodontology, Ribeirão Preto School of Dentistry, University of São Paulo, Ribeirão Preto, São Paulo, Brazil; 412619719487154859832 Instituto Grisi de Odontologia, Ribeirão Preto, São Paulo, Brazil; 451809894739967661899 Department of Stomatology, Public Health and Forensic Dentistry, Ribeirão Preto School of Dentistry, University of São Paulo, Ribeirão Preto, São Paulo, Brazil

## Abstract

Furcation defects pose a significant challenge in periodontal therapy. While regenerative approaches are generally preferred, resective procedures may remain essential depending on defect morphology and root configuration. This case series aims to demonstrate the clinical benefits of combining resective and regenerative techniques using periodontal microsurgery to treat maxillary molars with deep trifurcation defects. Three patients with extensive Class II trifurcation defects in maxillary molars, unresponsive to prior non-surgical periodontal therapy, were managed using a dental operating microscope and cone-beam computed tomography for accurate diagnosis and planning. The surgical protocol included selective buccal root resection alongside guided tissue regeneration using enamel matrix derivative, collagen membrane and heterologous bone graft. Short- and long-term post-operative follow-ups, including clinical and radiographic evaluations during supportive periodontal care, demonstrated tissue volume preservation and complete resolution of the infection. These cases indicate that, when appropriately selected, the combined microsurgical resective-regenerative approach offers a predictable, minimally invasive treatment option for advanced Class II trifurcation defects in maxillary molars, providing a valuable alternative to extraction and implant placement.

## Key points

- Microsurgical principles with combined resective-regenerative techniques for advanced Class II trifurcation defects in maxillary molars are rarely reported in the literature.
- Strategic buccal root resection improves access and defect configuration while preserving remaining palatal support.
- Success depends on accurate diagnosis with advanced imaging, careful case selection, minimally invasive microsurgical approach, and strict adherence to post-operative protocols.
- Main limitations include anatomical complexity, defect extent and configuration, and the technical sensitivity of procedures requiring high surgical expertise and precision.

## Introduction

Interradicular osseous defects occurring between the roots of multirooted teeth pose a significant challenge in periodontology. These defects may result from disease or trauma, and are classified as bifurcation or trifurcation defects, depending on the number of roots involved. Their prevalence appears to be similar across genders but are most commonly observed in maxillary molars.^1^^,^^2^

Diagnosis is based on the extent of horizontal bone loss, which guides treatment planning. Class I defects (<3 mm) usually respond to non-surgical periodontal therapy (NSPT). However, Class II defects (≥3 mm without complete furcal involvement) and Class III defects (complete through-and-through involvement) are significantly more challenging, particularly trifurcations in maxillary molars.^3^ In these cases, the confined interradicular space limits both instrumentation and visibility, often necessitating surgical intervention.^4^

Surgical treatment may follow regenerative or resective strategies. Regenerative techniques, favoured in the literature,^5^ including guided tissue regeneration (GTR), aim to restore periodontal structures – cementum, periodontal ligament, and alveolar bone – using membranes, grafts, and biologic mediators.^6^^,^^7^^,^^8^ However, when defect morphology limits surgical access or the potential for regeneration, resective procedures such as root resection and tunnelling become necessary.^9^ Although these procedures facilitate effective hygiene and long-term maintenance, they may result in some tissue volume loss.^10^

In this context, minimally invasive periodontal microsurgery – incorporating magnification, microsurgical instruments, and delicate techniques – enhances surgical precision, reduces tissue trauma, and improves wound stability, thus increasing the predictability of treatment outcomes.^10^^,^^11^ This case series aims to illustrate the benefits of combining resective and regenerative microsurgical techniques in the treatment of maxillary molars with deep Class II trifurcation defects.

## Case presentations

Three representative cases of trifurcation defects in maxillary molars resulting from periodontal disease were selected from patients treated at a private endodontics and periodontics practice (Paulínia, São Paulo, Brazil) between October 2020 and May 2025. Clinical data and images were obtained from the patients' electronic health record and the image database. Risk assessments were discussed among the treating dentists and the patients.

All patients underwent microsurgical procedures performed under a dental operating microscope (OPMI MD, Zeiss, Oberkochen, Germany), equipped with high-resolution photography accessories. Following local infiltrative anaesthesia, a buccal triangular mucoperiosteal flap was elevated to achieve complete exposure of the cortical bone. Osteotomy was performed using surgical burs and manual micro chisels, and root resection was carried out with ultrasonic tips. After thorough curettage of granulomatous tissue and inspection of the alveolus, guided tissue regeneration was performed using EDTA 24% (PrefGel, Straummann, Basel, Switzerland) and enamel matrix derivative (Emdogain, Straummann, Basel, Switzerland) for root surface conditioning, heterologous bone graft (Bio-Oss Small, Geistlich, Wolhusen, Switzerland) placement using a bone curette, and positioning of a collagen membrane (Bio-Gide, Geistlich, Wolhusen, Switzerland) without fixation methods. Sling 6-0 polypropylene microsutures were placed along the sulcular margin, and interrupted sutures were used for the vertical releasing incision.

Post-operative care included the use of an antimicrobial mouth rinse (0.12% chlorhexidine, twice a day for a week), analgesics (dipyrone 1000 mg, three times a day for three days), and anti-inflammatory medication (dexamethasone 4 mg, twice a day for two days in Cases 1 and 2, or naproxen 500 mg, twice a day for five days in Case 3). Antibiotic therapy consisted of azithromycin 500 mg for five days, starting one hour prior to surgery. Additionally, photobiomodulation (Therapy XT, DMC, São Carlos, Brazil) was applied daily for the first three days (808 nm and 660 nm, 100 mW, 2J per point, four points), followed by weekly sessions for one month (660 nm, 100mW, 2J per point, four points).

In each case, informed consent was obtained from the patient for publication of the case details. This case series has been reported in line with the PROCESS Guideline^12^ and received approval from the institutional ethics committee (CAAE: 91922825.4.0000.5419).

### Case 1

**Fig. 1:**
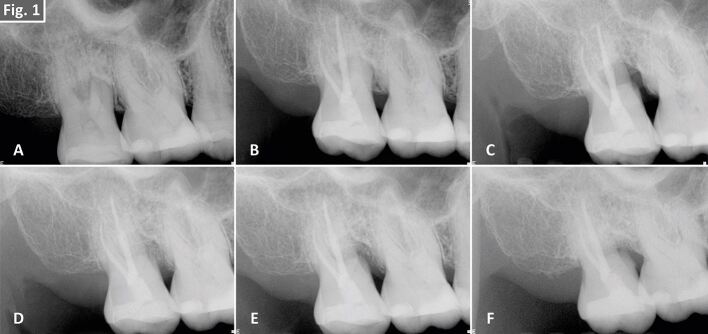
Case 1: Periapical radiograph showing a radiolucent lesion in the furcation region of tooth 17 (A) and post-root canal treatment (B). Intra-operative radiograph following the mesiobuccal root resection of tooth 17 (C). Immediate postoperative radiograph after guided tissue regeneration (D). Radiographs at one month (E) and four years (F), demonstrating bone remodelling and tooth preservation

A 55-year-old female patient was referred for evaluation of an interradicular radiolucent area in tooth 17, identified on a routine radiograph, with no reported complaint. The patient was asymptomatic, and clinical and radiographic examinations revealed excellent oral health and biofilm control. Tooth 17 exhibited a furcation radiolucency (Fig. 1A), a positive pulp vitality response, Grade I mobility, bleeding on probing, occlusal trauma, a probing depth of 8 mm in the mesiopalatal furcation and no evidence of purulent exudate. Her medical history revealed no systemic condition.

**Fig. 2:**
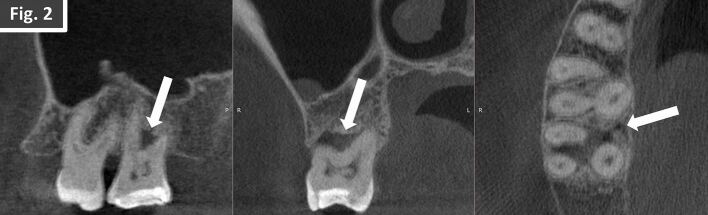
Case 1: Initial CBCT examination (sagittal, coronal, axial), showing a hypodense lesion in the furcation region of tooth 17 (arrows), suggestive of a periodontal infection

Initial treatment consisted of occlusal adjustments and full-mouth dental prophylaxis, followed by NSPT on tooth 17, performed under magnification, with the use of enamel matrix derivative. The patient was then placed on regular supportive periodontal care (SPC) every three months. After two years of SPC, the patient showed reduced bleeding and probing depth, along with cessation of tooth mobility; however, radiographic evaluation showed persistence of the lesion. Consequently, a cone-beam computed tomography (CBCT) scan was performed, showing an extensive Class II furcation lesion involving the trifurcation region of the roots of tooth 17 (Fig. 2), as well as a small amount of calculus on the mesiobuccal root. Considering the patient's optimal oral health and her desire to retain the affected tooth, a conservative surgical approach was pursued, consisting of mesiobuccal root resection combined with furcation regenerative therapy to improve the prognosis.

**Fig. 3:**
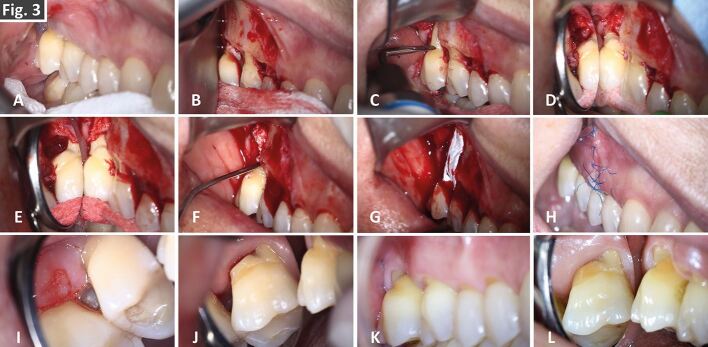
Case 1: Pre-operative buccal view (A). Cortical bone exposure (B). Mesiobuccal root resection of tooth 17 with an ultrasonic tip (C). Exposure of granulomatous tissue in the furcation region of tooth 17 (mirror view) (D). Buccal view after furcation debridement (mirror view) (E). Placement of enamel matrix derivative and bone graft (F). Collagen membrane over graft (G). Immediate post-operative buccal view without mirror (H) and with mirror at one week (I) and one month (J) post-operatively, respectively. Buccal views without mirror (K) and with mirror (L) at four years post-operatively, showing excellent sealing of furcation region

Endodontic treatment was completed for tooth 17 (Fig. 1B), performed pre-emptively in anticipation of the mesiobuccal root resection to facilitate surgical access. During the microsurgical procedure (Fig. 1C–D and Fig. 3A–H) granulomatous tissue was observed in the trifurcation area. After root resection and debridement of the remaining roots, biomaterials for GTR were placed (Fig. 3F–G). Short- and long-term follow-ups (four years) showed progressive and complete tissue repair, resolution of periodontal infection, furcation closure, and absence of mobility (Fig. 1E–F and Fig. 3I–L).

### Case 2

**Fig. 4:**
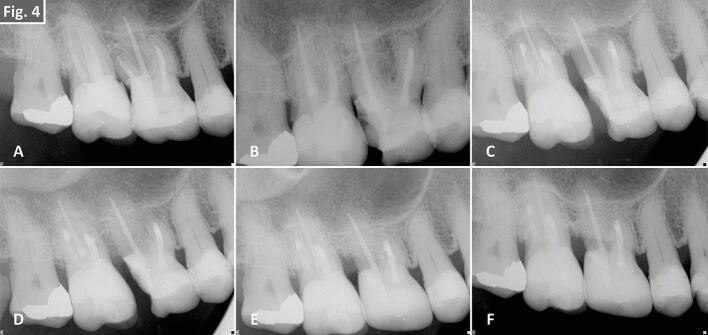
Case 2: Periapical radiograph showing a radiolucent lesion surrounding the distobuccal root and furcation region of tooth 16 (A). Periapical radiograph after selective palatal root canal retreatment of tooth 16 (B). Intra-operative radiograph following the distobuccal root resection of tooth 16, along with apicoectomy, retropreparation and retrofilling of the remaining buccal canals of teeth 17 and 16 (C). Immediate postoperative radiograph after guided tissue regeneration (D). Radiograph four months (E) and 27 months (F), highlighting sustained healing and bone regeneration

**Fig. 5:**
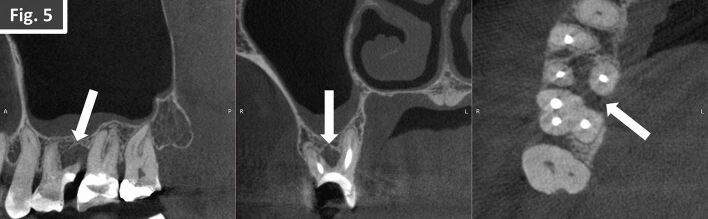
Case 2: Initial CBCT examination (sagittal, coronal, axial), showing a large interradicular hypodense lesion within the furcation region of tooth 16 (arrows), suggestive of periodontal infection. Thickening of the sinus membrane is also evident

A 64-year-old female patient was referred due to a radiolucent lesion around the distobuccal root of tooth 16. Upon clinical, radiographic and tomographic evaluation, tooth 16 presented a very large Class II furcation lesion, bleeding on probing, and a probing depth of approximately 9 mm, with no evidence of tooth mobility (Fig. 4A and Fig. 5). Teeth 17 and 16 had a history of endodontic treatment. The patient was asymptomatic, exhibited generally good oral health and reported no systemic conditions.

**Fig. 6:**
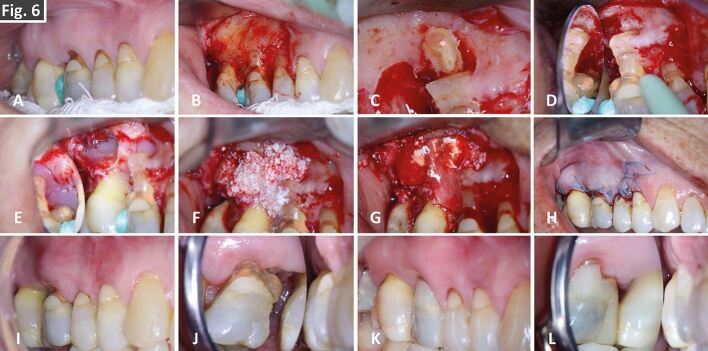
Case 2: Pre-operative buccal view (A). Cortical bone exposure (B). Mesiobuccal apex of tooth 16, post-apicoectomy, showing an untreated MB2 canal (C). Buccal view after distobuccal root resection, mesiobuccal root retrofilling, and root/furcation debridement of tooth 16 (mirror view) (D). Placement of enamel matrix derivative (mirror view) (E). Bone graft placement (F). Collagen membrane over graft (G). Immediate postoperative view (H). Buccal view at two weeks postoperatively without mirror (I) and with mirror (J), showing soft tissue closure, albeit distant from root end. Buccal view at 27 months post-operatively without mirror (K) and with mirror (L), showing complete sealing of furcation region, in contrast to Fig. 6J

Initial treatment included full-mouth dental prophylaxis, NSPT in the upper right quadrant, followed by SPC every four months. After three years, the patient was indicated for restorative procedures on teeth 17 and 16; however, due to persistent infection, surgical intervention was recommended prior to the restorative phase. Selective endodontic retreatment was performed on the palatal root for tooth 17 (Fig. 4B) followed by a microsurgical procedure (Fig. 4C–D and Fig. 6A–H), which included distobuccal root resection of tooth 16, and endodontic microsurgery (apicoectomy/retropreparation/retrofilling) on the remaining buccal roots of teeth 17 and 16. Following GTR (Fig. 6E–G), the vestibular flap was apically split to allow slight coronal advancement. A provisional restoration in the interproximal area was immediately placed to aid in biomaterial retention and to support stabilisation of the transient post-surgical tooth mobility.

The patient showed both radiographic and clinical resolution of the infection (Fig. 4E–F and Fig. 6I–L). At 27 months post-operatively, she remains asymptomatic, with good biofilm control, preservation of tissue volume, and a healthy, restored periodontal status.

### Case 3

**Fig. 7:**
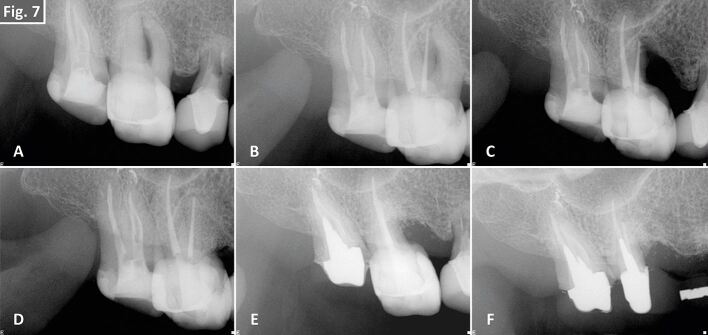
Case 3: Periapical radiograph showing a radiolucent lesion in the furcation area of tooth 16, with caries beneath restorations, missing tooth 15, and tooth 14 planned for extraction (A). Periapical radiograph after root canal treatment of tooth 16 (B). Intra-operative radiograph following the mesiobuccal root resection of tooth 16 (C). Immediate post-operative radiograph after guided tissue regeneration (D). Radiographs at eleven months (E) and three years (F)

A 59-year-old female patient was referred to our clinic for a routine examination, reporting pain in tooth 16. Clinical and radiographic evaluations revealed a vertical root fracture in this tooth (which was immediately indicated for extraction), as well as the need to replace several unsatisfactory restorations and prostheses. Additionally, and central to this report, a radiolucent area was detected in the furcation of tooth 16, along with the absence of tooth 15 and a root perforation of tooth 14 (Fig. 7A). The patient's medical history included anxiety and depression.

**Fig. 8:**
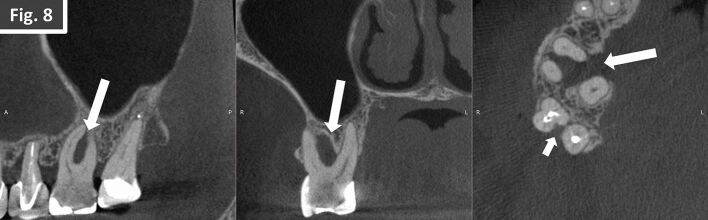
Case 3: Initial CBCT examination (sagittal, coronal, axial). Hypodense interradicular lesion (large arrows) associated with tooth 16, suggesting extensive mesiolingual Class II trifurcation involvement due to periodontal disease. Tooth 16 also exhibited a distobuccal Class II furcation lesion (axial slice, small arrow)

A CBCT scan (Fig. 8) was performed to assess the feasibility of conservative treatment for tooth 16. The scan showed large, isolated Class II furcation defects associated with teeth 17 and 16, as well as an intraosseous defect surrounding tooth 14. The latter was indicated for extraction, and although the prognosis for teeth 17 and 16 was poor, the patient expressed a strong preference for attempting preservation.

Initial steps included comprehensive dental prophylaxis, followed by the removal of unsatisfactory prostheses and restorations. Conventional endodontic treatment was successfully completed for tooth 16 (Fig. 7B), in preparation for root resection, while retreatment of the 17 was not indicated due to absence of periapical lesion in the CBCT examination.

**Fig. 9:**
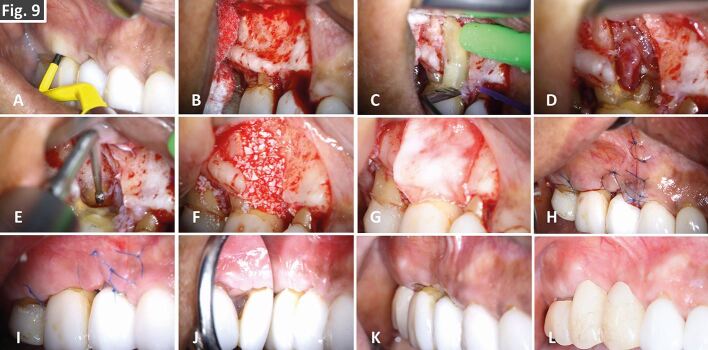
Case 3: Pre-operative buccal view (A). Buccal cortical bone of tooth 16 before osteotomy (B). Mesiobuccal root resection with an ultrasonic tip (C). Exposure of furcation region and granulomatous tissue (D). Furcation and root debridement with a diamond ultrasonic tip (E). Bone graft placement after root conditioning with enamel matrix derivative (F). Collagen membrane over the bone graft (G). Immediate postoperative view (H). Buccal views at one week (I), two weeks (mirror) (J), eleven months (K) and three years (L)

Despite urgent clinical recommendations, the patient returned only after 18 months to continue treatment. At this stage, a microsurgical approach was carried out, including mesiobuccal root resection of tooth 16, root debridement for teeth 17 and 16, complete removal of granulomatous tissue, and the placement of biomaterials for GTR (Fig. 9A–H). Tooth 16 initially exhibited grade I mobility, which transiently increased to Grade II during the immediate post-operative period, but fully resolved during long-term follow-up. Although a small amount of bone graft was lost in the initial healing phase, follow-ups demonstrated complete resolution of the infection, with preservation of both soft and hard tissue (Fig. 7E–F and Fig. 9I–L).

## Discussion

Class II and III furcation defects, particularly in maxillary molars, are strongly associated with increased risks of tooth loss due to their anatomical complexity, which limits access and reduces the effectiveness of non-surgical debridement, ultimately contributing to persistent inflammation and disease progression.^13^^,^^14^^,^^15^ Consequently, surgical intervention is often indicated for advanced furcation lesions, offering improved clinical outcomes.^16^^,^^17^ In light of these considerations, this case series describes a microsurgical approach for the management of deep Class II furcations in maxillary molars through the combined use of buccal root resection and guided tissue regeneration.

All patients required an interdisciplinary approach integrating periodontal, endodontic, and restorative principles.^18^ They had previously undergone non-surgical periodontal therapy without sufficient improvement, presenting with persistent pockets ≥6 mm, a condition previously associated with lower success rates of NSPT.^14^ They were non-smokers, systemically healthy, highly motivated and demonstrated good plaque control, all of which are well-established predictors of favourable regenerative outcomes.^16^^,^^19^ Following Jacobs *et al.* (2024), CBCT examination was used pre-operatively to assess bone architecture, root anatomy, and treatment history, thereby improving diagnostic accuracy and treatment planning.^20^

Historically, procedures such as tunnelling and root resection have been employed for advanced furcation defects. Tunnelling, typically used in mandibular molars, improves access for hygiene but requires ideal root anatomy and high patient compliance; otherwise, complications such as root caries may arise.^15^ Root resection involves removing a compromised root while preserving others to maintain function. Though technically demanding and associated with risks such as fractures and endodontic complications, root resection alone remains viable in carefully selected cases, including maxillary molars.^18^^,^^21^

In contrast to these traditional methods, regenerative approaches have gained prominence due to their superior clinical outcomes. GTR enhances bone fill and reestablishes periodontal architecture, becoming the preferred treatment over resective surgery or extraction for Class II in both mandibular and maxillary molars.^16^^,^^17^^,^^22^ However, depending on the depth and configuration of the lesion, access for regenerative therapy may also be restricted, necessitating the use of resective procedures to allow adequate treatment.^21^

In our cases, although the lesions extended palatally, the palatal roots showed favourable attachment levels and regenerative potential, which may explain the absent or mild tooth mobility observed at baseline and justified their preservation. The buccal roots, however, were also compromised and contributed to the complexity of the defect. Their strategic removal facilitated surgical access and visibility and simplified the defect configuration. Tooth mobility increased slightly in the immediate postoperative period – likely due to surgical manipulation and transient periodontal support alterations – but resolved completely during long-term follow-up. To further support healing and regenerative success, and to avoid potential drawbacks such as soft tissue recession, papilla loss, and post-operative bone resorption – which are inherent to flap elevation – the protocol incorporated microsurgical principles. Magnification, micro instruments, minimal incisions, and primary wound closure are associated with reduced trauma and better soft tissue preservation.^10^^,^^23^ Additionally, photobiomodulation was applied post-operatively to enhance healing and minimise discomfort, and the patients were enrolled in a structured SPC programme, which played an important role in maintaining outcomes.^19^^,^^24^^,^^25^

Ultimately, when combined with regenerative techniques, this minimally invasive approach aligned with our patients' values and expectations – particularly since advanced furcation defects are often asymptomatic and discovered incidentally.^26^^,^^27^ Treatment proposals involving tooth extraction or complex rehabilitations may cause emotional distress and hesitation.^28^^,^^29^ When appropriately selected and managed, preserving compromised teeth has proven to be a valuable alternative to dental implants in posterior regions, especially considering the potential risks of peri-implantitis, extended healing periods, higher overall treatment costs, and the comparable long-term survival rates.^30^^,^^31^

## Conclusions

This case series demonstrates that a conservative microsurgical approach combining selective buccal root resection with guided tissue regeneration can effectively manage advanced Class II trifurcation defects in maxillary molars. Careful case selection, interdisciplinary collaboration, incorporation of microsurgical principles, and consistent maintenance care were essential for achieving positive outcomes. This minimally invasive approach may offer a valuable alternative to extraction and implant therapy, aligning with patient preferences and preserving natural dentition whenever feasible.
